# Supplementary material for: The Value of Magnetic Resonance Diffusion-Weighted Imaging and Dynamic Contrast Enhancement in the Diagnosis and Prognosis of Treatment Response in Patients with Epithelial Serous Ovarian Cancer
Source: Cancers (Basel). 2022 May 17;14(10):2464. doi: 10.3390/cancers14102464 (PMC9139226; doi:10.3390/cancers14102464)
Supplement: Supplementary file 1 [file cancers-14-02464-s001.zip › cancers-1713950-supplementary.pdf]

Table S1. The complete MR study protocol.

Parameter	T2 TSE	T2 TSE Fat-Sat	DW EPI	T2 TIRM	Vibe 3D T1 GRE	T1 GRE (In- and Outphase)	T1 TSE Fat-Sat	T2 TSE (BLADE) Fat-Sat (SPAIR)
Repetition time [ms]	4250	2110	3800	6100	3,05	125	510	2300
Echo time [ms]	117	123	73	39	1,13	1: 2,22<br>2: 4,92	9,6	116
Flip angle [deg.]	137	150	90	150	10	70	150	150
iPAT factor	-	2	2	-	2	2	-	2
Plane	axial,sagital coronal	axial	axial	axial	axial	axial	axial,sagital coronal	axial, coronal
Number of signal averages	1	1	4	1	1	1	1	1
Field of view - FOV [mm]	360	360	360	360	360	360	360	360
Rectangular FOV [%]	75,100, 100	100	75	75	75	75	75	100
Breath-hold	No	No	No	No	No	No	No	Yes
Resolution (mm)	0,7x0,7x5	1,4x1,4x5	B value: 0,50,500,1000,1500	0,9x0.9x5	1,7x1,3x3	1,3x1,3x5	0,9x0,9x5	1,4x1x4x6
